# Genome Sequence of an Emerging *Salmonella enterica* Serovar Infantis and Genomic Comparison with Other *S*. Infantis Strains

**DOI:** 10.1093/gbe/evaa048

**Published:** 2020-04-06

**Authors:** Emiliano Cohen, Galia Rahav, Ohad Gal-Mor

**Affiliations:** e1 The Infectious Diseases Research Laboratory, Sheba Medical Center, Tel-Hashomer, Israel; e2 Sackler Faculty of Medicine, Tel Aviv University, Israel; e3 Department of Clinical Microbiology and Immunology, Tel Aviv University, Israel

**Keywords:** *Salmonella* Infantis, WGS, pESI, mobile genetic elements, HGT, virulence

## Abstract

*Salmonella enterica* serovar Infantis (*S*. Infantis) is one of the dominant serovars of the bacterial pathogen *S. enterica*. In recent years, the number of human infections caused by *S*. Infantis has been increasing in many countries, and often the emerging population harbors a unique virulence-resistant megaplasmid called plasmid of emerging *S*. Infantis (pESI). Here, we report the complete gap-free genome sequence of the *S*. Infantis Israeli emerging clone and compare its chromosome and pESI sequences with other complete *S*. Infantis genomes. We show a conserved presence of the *Salmonella* pathogenicity islands 1–6, 9, 11, 12, and CS54 and a common integration of five bacteriophages in the *S*. Infantis chromosome. In contrast, we found variable presence of additionally three chromosomally integrated phages and eight modular regions in pESI, which contribute to the genetic and phenotypic diversity (including antimicrobial resistance) of this ubiquitous foodborne pathogen.

## Introduction

The abundant foodborne pathogen *Salmonella enterica* (*S. enterica*) is a Gram-negative, highly diverse bacterium that can infect and colonize a broad array of animal and human hosts. This single bacterial species comprises of >2,600 antigenically distinct serovars that can be classified according to their host-specificity and their occasioned disease (Gal-Mor 2019).

Non-typhoidal serovars (NTS) like *Salmonella enterica* serovar Typhimurium (*S*. Typhimurium) or *S. enterica* serovar Infantis (*S*. Infantis) are known to possess a wide host-specificity and are capable of infecting various animal species including reptiles, birds, and mammals. In immunocompetent humans, infection with NTS serovars normally provokes a self-limiting localized inflammation of the terminal ileum and colon, called gastroenteritis. The assessed annual global burden of gastroenteritis caused by NTS infections is 78.7 million incidents, resulting in 59,000 deaths (Havelaar et al. 2015).

Amongst >2,600 *S. enterica* serovars known to date, *S*. Infantis is one of the most prevalent serovars worldwide. In the United States, *S*. Infantis was ranked sixth, in the occurrence hierarchy (Crim et al. 2015) and in the European Union, *S*. Infantis was rated third in the prevalence order, following serovars Enteritidis and Typhimurium (ECDC 2014). Moreover, in recent years, *S*. Infantis is the most frequently reported *S. enterica* serovar from food-producing animals (mainly from the poultry production chain) and various food products in Europe (EFSA 2017).

Latterly, we demonstrated that serovar Infantis is largely associated with infections of infants younger than two years old and adheres better to host cells than the serovar Typhimurium. Nevertheless, in comparison to *S*. Typhimurium, *S*. Infantis was shown to be less invasive in humans and causes lower inflammation in the colitis mouse model. These differences were attributed to lower expression levels of the *Salmonella* pathogenicity island (SPI) 1 genes in *S*. Infantis compared with *S*. Typhimurium (Aviv et al. 2019).

In Israel, a rapid and clonal emergence of *S*. Infantis was reported in 2010, and from 2008 to 2015 *S*. Infantis was the most dominant serovar isolated from both human and poultry sources (Gal-Mor et al. 2010; Aviv et al. 2014). Noteworthy, the emergence of *S*. Infantis has been further reported in multiple countries around the world including Germany (Hauser et al. 2012), France, Belgium (Cloeckaert et al. 2007), Hungary (Nogrady et al. 2008), Russia (Bogomazova et al. 2020), Honduras (Liebana et al. 2004), Japan (Shahada et al. 2010), and Australia (Ross and Heuzenroeder 2008), indicating that *S.* Infantis is a globally emerging serovar and a primary source of poultry infection and human salmonellosis. A recent study addressing the genetic structure of the global *S*. Infantis population has shown that *S*. Infantis is a polyphyletic serovar and has evolved in three separate lineages, with specifically one dominant emerging lineage (Gymoese et al. 2019).

Previously, we have reported that the fast and clonal *S*. Infantis emergence was facilitated by lateral acquisition of a novel virulence-resistance megaplasmid, designated pESI (standing for plasmid of emerging *S*. Infantis) that contributes to multidrug resistance and enhanced pathogenicity of pESI-positive strains (Gal-Mor et al. 2010; Aviv et al. 2014). We specifically showed that pESI encodes several virulence factors, including the yersiniabactin—iron acquisition system, as well as the Klf and Ipf chaperon-usher fimbriae. Furthermore, this plasmid carries various mobile elements encoding antibiotic and mercury resistance genes and at least three independent toxin/antitoxin systems (MazEF/PemKI, CcdAB, and VagCD) (Aviv et al. 2014, 2016, 2017).

Subsequently, genetically related pESI-like plasmids were also found in additional emergent *S*. Infantis strains in Spain (Iriarte et al. 2017), Switzerland (Hindermann et al. 2017), Italy (Franco et al. 2015), Hungary (Szmolka et al. 2018), Japan (Yokoyama et al. 2015), the USA (Tate et al. 2017), and Russia (Bogomazova et al. 2020). These findings indicate worldwide dissemination of *S*. Infantis strains harboring pESI-like megaplasmids that play an important role in the evolution and epidemiology of globally emerging *S*. Infantis lineages.

Here, we report the complete and gap-free genome sequence of the emerging *S*. Infantis clone, represented by the 119944 Israel-isolated strain and present genomic analysis and comparison with other complete genomes of this serovars. Our results demonstrate a conserved distribution of 10 SPIs and five chromosomal prophages integrated into the genome of *S*. Infantis. Furthermore, we define core and variable regions in pESI and highlight the circulation of pESI-like plasmids among globally emerging *S*. Infantis strains.

## Materials and Methods

### Whole-Genome Sequencing

Genomic DNA from *S*. Infantis strain 119944, as a representative isolate of the emergent *S*. Infantis population in Israel (Gal-Mor et al. 2010) was isolated using the GenElute Bacterial Genomic DNA Kit (Sigma–Aldrich). Whole-genome sequencing that was performed at the Technion Genomic Center of the Israeli Institute of Technology (Haifa, Israel) has generated 16 × 10^6^ paired‐ends shorts reads by an Illumina Genome Analyzer IIx platform (Illumina, Inc.) and 291,510 long reads using a MinION sequencer (Oxford Nanopore Technologies). The average MinION reads length was 11,538 bp and the N50 was 28,095 bp long. The quality of the short Illumina and the long MinIon reads (fastq files) was evaluated using FastQC (version 0.11.5) and NanoPlot tools, respectively.

### Genome Assembly

Both the short (Illumina) and long (MinION) reads were combined for hybrid de novo assembly using the Unicycler (version 0.4.8-beta) pipeline (Wick et al. 2017a, 2017b). Unicycler assembler employed SPAdes (version 3.13) with error correction and automatic selection of k-mer length to produce short reads assembly graph (contigs). In the next step, the miniasm and Racon Unicycler’s modules were used for long reads and contigs assembly. The resulting assembly was then polished by pilon (version 1.22). The hybrid assembly of the *S.* Infantis 119944 genome resulted in two closed scaffolds corresponding to the chromosome (4,725,957 bp) and the pESI plasmid (285,081 bp), while the genome was covered 895×. The complete *S*. Infantis 119944 chromosome (accession number CP047881) and pESI (accession number CP047882) assembles were deposited in the NCBI database.

### Bioinformatics Analyses

Genome comparison of the *S*. Infantis 119944 chromosome and plasmid was done against all *S*. Infantis complete genomes found at the NCBI database including FSIS1502916 (assembly number GCA_001931575.1); FARPER-219 (GCA_006402875.1); FSIS1502169 (GCA_001931555.1); N55391 (GCA_001931595.1); CVM44454 (GCA_001931615.1); 1326/28 (GCA_000953495.1); NCTC6703 (GCA_900478235.1); and CFSAN003307 (GCA_002863785.1). Sequences alignment was conducted using Mauve (Rissman et al. 2009) and compared by BRIG (Alikhan et al. 2011). Phages and their integration sites were identified using PHASTER (Arndt et al. 2016). SPIs sequences were downloaded from the pathogenicity islands database (PAIDB) (http://www.paidb.re.kr/browse_pais.php?m=p#Salmonella enterica) and BLASTed against the assembled *S*. Infantis 119944 genome.

## Results and Discussion

To advance better understanding of the global epidemiology and genomics of *S*. Infantis we applied hybrid assembly while combining short reads from Illumina sequencing together with long reads from MinION platform. This approach allowed determining a complete gap-free genome sequence of *S*. Infantis isolate 119944 that was covered 895×. The complete genome of *S*. Infantis 119944 has a 53.2% GC content and composes of one circular 4,725,957 bp chromosome and a 285,081 bp plasmid, which we previously named pESI. Using the NCBI prokaryotic genome annotation pipeline (PGAP), we found that the *S*. Infantis 119944 genome encodes 4,853 genes, 4,612 proteins, 84 tRNA genes, and harbors122 pseudogenes.

To account for conserved and unique regions in the 119944 genome, the chromosome and the pESI plasmids were compared with eight *S*. Infantis complete genomes available at the NCBI database. Supplementary table 1, Supplementary Material online shows the main features and relevant metadata of the compered *S*. Infantis genomes. The *S*. Infantis genome size of these nine completely sequenced strains varied between 4,630,342 bp (strain NCTC6703) and 5,089,781 bp (FARPER-219) and harbored between none and two plasmids.

**Fig. 1.**
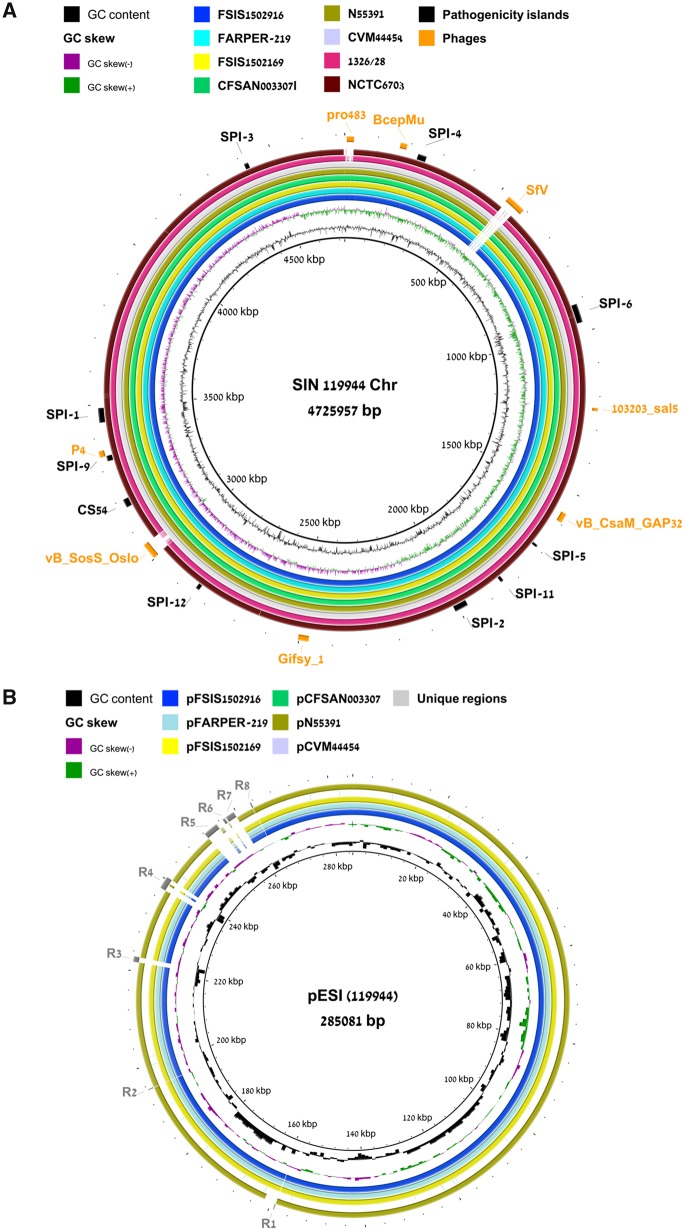
—Chromosome and pESI sequence comparison between *S*. Infantis strains. (*A*) The chromosome sequence of strain 119944 was compared with the complete chromosome sequence of eight *S*. Infantis strains (FSIS1502916; FARPER-219; FSIS1502169; N55391; CVM44454; 1326/28; NCTC6703; and CFSAN003307 using the BRIG tool [Alikhan et al. 2011]). The genome coordinates of the *S*. Infantis 119944 strain are shown by the internal black ring and the GC content is indicated by the black plot. The distribution of SPIs and prophage across the genomes are shown by black and orange boxes, respectively. (*B*) The DNA sequence of plasmids from six *S*. Infantis strains that were found to harbor megaplasmids (pFSIS1502916, pFARPER-219, pFSIS1502169, pN55391, pCVM44454, and pCFSAN003307) were compare to the sequence of pESI in 119944 using BRIG. Eight variable regions (R1–R8) that were found to be uniquely present in pESI in 119944 are indicated by the gray boxes.

The distribution of SPIs was constant across *S*. Infantis 119944 and the other compared genomes, and all of them were found to carry intact SPIs-1–6, SPI-9, SPI-12, and CS54 (fig. 1*A* and table 1). In addition, all of these *S*. Infantis genomes harbor a 9 kb shorter version of SPI-11, instead of the 15.7 kb island, known in *S*. Choleraesuis SC-B67 (Jacobsen et al. 2011). Nonetheless, all of the SPI-11-associated virulence genes are present in the *S*. Infantis SPI-11, including *pagC, pagD*, *msgA*, *envF*, and the T3SS effector gene *sopF*.

**Table 1:** Distribution of PAIs and Prophages in the *S*. Infantis 119944 Genome

Element	Name	Accession Number	Start	End	Length (bp)	Integrity	Unique (to 119944), Diverse or Conserved
Phage	pro483	NC_028943	6,016	27,791	21,776	Intact	Diverse
Phage	BcepMu	NC_005882	167,739	188,179	20,441	Incomplete	Conserved
Phage	SfV	NC_003444	529,849	589,113	59,265	Intact	Unique
Phage	103203 sal5	NC_031946	1,234,425	1,243,356	8,932	Incomplete	Conserved
Phage	vB CsaM GAP32	NC_019401	1,565,183	1,594,251	29,069	Incomplete	Conserved
Phage	Gifsy 1	NC_010392	2,471,266	2,503,308	32,043	Intact	Conserved
Phage	vB SosS Oslo	NC_018279	3,002,249	3,050,660	48,412	Intact	Diverse
Phage	P4	NC_001609	3,342,337	3,360,947	18,611	Incomplete	Conserved
PAI^a^	SPI-1	NC_006905_P5 (43.5 kb)	3,445,488	3,489,766	44,279	Intact	Conserved
PAI^a^	SPI-2	NC_006905_P3 (41.8 kb)	1,971,307	2,013,190	41,884	Intact	Conserved
PAI^a^	SPI-3	NC_006905_P6 (12.8 kb)	4,410,311	4,423,076	12,766	Intact	Conserved
PAI^a^	SPI-4	NC_006905_P7 (26.7 kb)	227,272	253,975	26,704	Intact	Conserved
PAI^a^	SPI-5	NC_006905_P1 (5.7 kb)	1,690,962	1,697,840	6,879	Intact	Conserved
PAI^a^	SPI-6	NC_003198_P1 (58.7 kb)	913,174	971,088	57,915	Intact	Conserved
PAI^a^	SPI-9	NC_003198_P4 (15.7 kb)	3,325,575	3,341,871	16,297	Intact	Conserved
PAI^a^	SPI-11	NC_006905_P2 (15.7 kb)	1,839,408	1,848,476	9,069	Incomplete	Conserved
PAI^a^	SPI-12	NC_006905_P4 (11.1 kb)	2,837,772	2,848,978	11,207	Intact	Conserved
PAI^a^	CS54	AF140550 (25.3 kb)	3,173,887	3,199,139	25,253	Intact	Conserved

^a^ SPIs sequences for comparison were downloaded from http://www.paidb.re.kr/browse_pais.php?m=p#Salmonella enterica.

*Salmonella enterica* serovar Infantis 119944 genome was found to possess eight bacteriophages in its chromosome (fig. 1*A* and table 1). Five of which (*Burkholderia cenocepacia* phage BcepMu; *Salmonella* Phage 103203 sal5; *Cronobacter* phage vB CsaM GAP32; Gifsy 1; and *Enterobacteria* phage P4) were present in all of the compared *S*. Infantis genomes. In contrast, a 59.2 kb Enterobacteria SfV phage (accession number NC_003444.1, spanning positions 529849–589113) was found only in the 119944 genome. Similarly, the 21.7 kb *Escherichia* phage pro483 (NC_028943, covering positions 6016–27791) and the 48.4 kb *Salmonella* phage vB SosS Oslo (NC_001609, integrated between positions 3002249 and 3050660) were found in only a subset of the *S*. Infantis genomes. These results show that while SPIs distribution is conserved among the tested *S*. Infantis genomes, bacteriophages repertoire is diverse and contributes significantly to the genetic diversification of *S*. Infantis strains.

Next, we compared the genetic similarity between the corresponding *S*. Infantis plasmids. Six out of the eight compared *S*. Infantis strains were found to harbor megaplasmids with size ranging from 178.2 to 316.1 Mb, whereas two *S*. Infantis strains (1326/28 and NCTC6703) did not carry any plasmid. Among the six *S*. Infantis megaplasmids found, only five were actually pESI-related (fig. 1*B*), carried by *S*. Infantis strains that were isolated between 2014 and 2017. These results are consistent with the notion that pESI plasmids are associated with emerging (recent) *S*. Infantis strains and thus far were not identified in older isolates (Aviv et al. 2014).

Despite very high sequence similarity between pESI-related plasmids that were isolated from different geographically regions, the pESI of strain 119944 was found to contain eight unique regions ranging in size between 166 and 3,079 bp (indicated as unique regions R1–R8 in fig. 1*B*), which were not present in any of the other pESI-like plasmids included in this cohort. These are modular regions comprising insertion sequences elements, transposases or hypothetical proteins found in various plasmids. Interestingly, instead of region 8 (R8), all other pESI-like plasmids contain a different mobile element (possibly a transposon that carries transposases and IS 6-like insertion sequences), encoding arsenic resistance genes cluster as well as the *bla*_CTX-M-65_ gene (coding for an extended-spectrum β lactamases), which are lacking in the 119944 pESI.

Another recent study (Gymoese et al. 2019) that have used incomplete genomes of 105 *S*. Infantis isolates identified 16 strains harboring a conserved pESI-like plasmids of ∼280–283 kb. None of these plasmids contains the *bla*_CTX-M-1_ or *bla*_CTX-M-65_ genes as reported in pESI-like ESBL-positive plasmids (Franco et al. 2015; Hindermann et al. 2017; Tate et al. 2017). These differences highlight the modular nature of pESI and its genetic plasticity facilitated by its ability to “mix and match” mobile genetic elements and integrate them into a conserved pESI backbone. Moreover, because the ESBL-positive pESI-like plasmids were isolated from *S*. Infantis strains in USA (Tate et al. 2017), Peru (Vallejos-Sanchez et al. 2019), Switzerland (Hindermann et al. 2017), and Italy (Franco et al. 2015), it is highly possible that these pESI derivatives are globally disseminated.

In summary, we applied a state-of-the-art hybrid assembly approach and determined a gap-free complete sequence of a *S*. Infantis emerging clone that harbors the virulence-resistance megaplasmid pESI. By a conservative genomic comparison with other complete *S*. Infantis genomes, we defined core presence of ten SPIs and five prophages and identified conserved and variable regions in the pESI plasmid. We showed that the genetic and phenotypic diversity (especially antimicrobial resistance) of emerging *S*. Infantis strains is shaped by a varying repertoire of chromosomal prophages and integration of different mobile genetic elements into a conserved pESI backbone.

## Supplementary Material

Supplementary data are available at *Genome Biology and Evolution* online.

## Supplementary Material

evaa048_Supplementary_Data
