# Supplementary material for: Genome Sequence of an Emerging Salmonella enterica Serovar Infantis and Genomic Comparison with Other S. Infantis Strains
Source: Genome Biol Evol. 2020 Apr 6;12(3):223–8. doi: 10.1093/gbe/evaa048 (PMC7144548; doi:10.1093/gbe/evaa048)
Supplement: evaa048_Supplementary_Data [file evaa048_supplementary_data.docx]

**Supplementary Table 1. *S*. Infantis complete genomes included in this study**

| **Strain** | **Assembly** | **Source; location; and year of isolation** | **Genome Size (bp)** | **GC%** | **Chr size** | **Chr accession number** | **Plasmid size** | **Plasmid accession number** | **Sequencing technology** | **Reference** |
| --- | --- | --- | --- | --- | --- | --- | --- | --- | --- | --- |
| FSIS1502916 | GCA_001931575.1 | Poultry; USA NJ; 2015 | 5,050,625 | 52.18 | 4,728,107 | CP016408.1 | 322,518 | CP016409.1 | PacBio | ([Tate, et al. 2017](#_ENREF_2)) |
| FARPER-219 | GCA_006402875.1 | Poultry; Peru; 2017 | 5,089,781 | 52.04 | 4,727,696 | CP038507.1 | 320,892 | CP038508.1; CP038509.1 | PacBio RSII | ([Vallejos-Sanchez, et al. 2019](#_ENREF_3)) |
| FSIS1502169 | GCA_001931555.1 | Poultry; USA NC; 2015 | 5,050,248 | 52.18 | 4,727,126 | CP016406.1 | 323,122 | CP016407.1 | PacBio | ([Tate, et al. 2017](#_ENREF_2)) |
| N55391 | GCA_001931595.1; | Poultry; USA TN; 2014 | 5,043,928 | 52.18 | 4,727,114 | CP016410.1 | 316,814 | CP016411.1 | PacBio | ([Tate, et al. 2017](#_ENREF_2)) |
| CVM44454 | GCA_001931615.1 | Human; USA MA; 2014 | 5,043,245 | 52.18 | 4,727,085 | CP016412.1 | 316,160 | CP016413.1 | PacBio | N/A |
| 1326/28 | GCA_000953495.1 | Poultry; UK; 1973 | 4,710,675 | 52.30 | 4,710,675 | LN649235.1 | - | - | N/A | ([Olasz, et al. 2015](#_ENREF_1)) |
| NCTC6703 | GCA_900478235.1 | Human; USA CT; 1940 | 4,630,342 | 52.30 | 4,630,342 | LS483479.1 | - | - | N/A | ([Wheeler and Borman 1943](#_ENREF_4)) |
| CFSAN003307 | GCA_002863785.1 | creek water; USA VA; 2011 | 4,716,126 | 52.30 | 4,537,888 | CP019202.1 | 178,238 | CP019203.1 | PacBio | N/A |
| 119944 | SUB6837264 (submission #) | Human; Israel; 2008 | 5,011,038 | 52.3 | 4,725,957 | CP047881 | 285,081 | CP047882 | Illumina GAII and Oxford Nanopore MiniION | This study |

REFERENCES

Olasz F, et al. 2015. Genome Sequences of Three Salmonella enterica subsp. enterica Serovar Infantis Strains from Healthy Broiler Chicks in Hungary and in the United Kingdom. Genome Announc 3. doi: 10.1128/genomeA.01468-14

Tate H, et al. 2017. Comparative Analysis of Extended Spectrum Beta- Lactamase CTX-M-65-Producing Salmonella Infantis Isolates from Humans, Food Animals, and Retail Chickens in the United States. Antimicrob Agents Chemother. doi: 10.1128/AAC.00488-17

Vallejos-Sanchez K, et al. 2019. Whole-Genome Sequencing of a Salmonella enterica subsp. enterica Serovar Infantis Strain Isolated from Broiler Chicken in Peru. Microbiol Resour Announc 8. doi: 10.1128/MRA.00826-19

Wheeler KM, Borman EK 1943. Two New Salmonella Serotypes Isolated from Man. J Bacteriol 46: 481.
